# Psychometric Properties of the Chinese Revision of the Pitt Wellness Scale for People in the University Environment

**DOI:** 10.3389/fpsyg.2022.899880

**Published:** 2022-05-12

**Authors:** Xiangru Yan, Ye Gao, Hui Zhang, Chunguang Liang, Haitao Yu, Liying Wang, Sisi Li, Yanhui Li, Huijuan Tong

**Affiliations:** ^1^Department of Nursing, Jinzhou Medical University, Jinzhou, China; ^2^Department of Nursing, Liaoning University of Technology, Jinzhou, China; ^3^Department of Nursing, Panjin Vocational and Technical College, Panjin, China; ^4^Department of Stomatology, Shandong Medical College, Linyi, China; ^5^Department of Nursing, Shenyang Medical College, Shenyang, China

**Keywords:** well-being, Chinese university, Pitt Wellness Scale, validation, psychometric properties

## Abstract

**Background:**

The number of students enrolled in higher education in China accounts for more than one-fifth of the world, and universities, as a community of faculty, staff and scholars, currently do not have a scale that specifically assesses the well-being of the population in the environment of Chinese universities. However, the University of Pittsburgh has developed a comprehensive well-being scale, referred to as the Pitt Wellness Scale, specifically to measure people’s well-being in a university environment.

**Aims:**

Investigate the psychometric properties of the Pitt Wellness Scale in Chinese university environmental samples.

**Methods:**

The original scale was culturally adapted and modified through expert consultation, a random sample of 1870 current faculty, staff, and students were selected for the questionnaire survey. Exploratory factor analysis (EFA) and confirmatory factor analysis (CFA) were used to investigate the potential factor structure of the Chinese Revision of the Pitt Wellness Scale and to measure its reliability and validity. Finally, the factors that affect people’s well-being in the Chinese university environment were explored.

**Results:**

The Chinese Revision of the Pitt Wellness Scale retained 30 items, and the EFA supports a five-factor structure, which differed from the results of the original scale, and the CFA results showed that the model fitted well. The discriminant validity of the modified Chinese scale was excellent. The overall Omega coefficient of the scale was 0.958, and the reliability of the retest after 4 weeks was 0.821.

**Conclusion:**

The Chinese Revision of the Pitt Wellness Scale possesses satisfactory psychometric properties, and it can be considered an instrument for assessing personal well-being in Chinese university environment.

## Introduction

Well-being is different from happiness because it is a higher-level concept that reflects a more stable condition of being well, feeling satisfied, and being contented (Lindert et al., 2015). In 1946, the World Health Organization explicitly linked health and well-being, and Dutch scholars described well-being as an individual’s sense of wellness (Constitution of the world health organization, 1946; Cools et al., 2020). In 1967, Wilson described well-being as “being young, healthy, well-educated, having a high income, being outgoing, optimistic, untroubled, religious, having high self-esteem, being married, having a work ethic, having moderate expectations, as well as being knowledgeable, and it has nothing to do with gender” (Wilson, 1967).

Nationally and internationally, there is growing concerned about mental health and well-being, especially the well-being of young people (Eckersley, 2011; Hartwell, 2015). University students are a unique group within the young population with high levels of intelligence, ambition, and self-esteem, and students in higher education are a high-risk group for health problems (Lew et al., 2019). For another, the people in the university environment (faculty, staff, and students) are considered to be the group focused on higher education and research, the hope of the nation, and the future of the country. Therefore, as a place where new technologies, new ideologies, and advanced human resources are cultivated, it is particularly important to assess the overall well-being of people in the university environment.

According to an American study from the 2019 National College Health Assessment, 27.1% of students consider themselves to be in a high-stress state; 23.5% regarded anxiety, and 73.6% consulted a psychiatrist; 21.8% reported depression harmed their academic achievement (American College Health Association, 2017). The stress of transitioning from adolescence to adulthood, coupled with pressure from all aspects of school, can contribute to a decline in both the health and well-being of students. Universities should take responsibility for the health and well-being of their students. For faculty and staff, people’s well-being and work involvement are both positively correlated and mutually influenced (Shimazu and Schaufeli, 2009; Shimazu et al., 2012; Upadyaya and Salmela-Aro, 2013). Researches show that many Chinese teachers suffer from a high degree of stress, professional burnout, and high rates of turnover (Chan, 1998; Chan and Hui, 1995; Hui, 1998; Liu and Onwuegbuzie, 2012; Liu and Zhou, 2016; Wang et al., 2020). The level of well-being of faculty members affects not only their commitment to teaching but also the quality of education and even whether they stay in their educational positions. Hence, investigations into the well-being of people in university settings and timely interventions are imperative. However, no scale has been developed that can measure the well-being of people in university environments.

Many quality of life scales have been developed to attempt to measure well-being, but the quality of life does not necessarily represent a person’s level of well-being, and well-being should be viewed as a separate outcome rather than a component that is often included in quality of life measures (Diener et al., 1985; Pavot et al., 1998; Cools et al., 2020). Quality of life refers to the external quality of life, for example, environment or performance, while well-being is the individual’s subjective feeling of enjoying the internal quality of life (Veenhoven, 2001). When measuring the quality of life, it is uncertain whether intrinsic factors affect well-being due to the presence of extrinsic factors (Veenhoven, 2001). Well-being is a valuable concept that can be considered a separate measure from quality of life (Diener and Seligman, 2004), and it goes far beyond the traditional indicators that predict quality of life. Thus, using quality of life scales to measure a person’s well-being is biased.

There are many dimensions of well-being, and it is difficult to generalize well-being through a few simple dimensions. A review examined 99 indicators of well-being, of which 196 dimensions of well-being were identified, with dimensions focused on six key topic areas: mental well-being, social well-being, physical well-being, spiritual well-being, activity and functioning, and personal circumstances (Linton et al., 2016). Tom Rath and Jim Harte outlined five elements of well-being, which are career well-being, social well-being, financial well-being, physical well-being, and community well-being (Rath et al., 2010). Other research related to well-being includes other areas such as education, environment, the standard of living, etc. (Self and Statistics, 2014; Smale and Hilbrecht, 2014). The 2015 international assessment system Program for International Student Assessment (PISA) incorporated a new instrument that proposes five dimensions: cognitive, psychological, social, physical, and material, based on the PISA theoretical framework (Govorova et al., 2020). The instrument primarily assesses factors of well-being that significantly affect students’ academic performance and does not apply to the assessment of the overall well-being of university students.

Well-being scales have been less developed in China. British scholars Tennant et al. developed and validated the Warwick Edinburgh Mental Well-being Scale (WEMWBS), and in 2019 mainland China localized the WEMWBS with a sample population of mainland Chinese university students. This scale was developed for the general population, not just college students (Tennant et al., 2007; Fung, 2019). Yan Zhang and Richard Carciofo validated the College Student Subjective Wellbeing Questionnaire (CSSWQ), which assessed four more limited aspects of well-being related to academics (academic satisfaction, academic efficacy, school connectedness, and college appreciation) (Renshaw, 2018; Zhang and Carciofo, 2021). Other scales developed, such as the Oxford Happiness Inventory (Hills and Argyle, 1998) and General Well-Being Schedule (GWBS) (Fazio, 1977), are helpful for large-scale assessment of the general well-being status of the population, but there is no way to accurately capture the well-being of a specific population. However, Dr. Leming Zhou and Dr. Parmuto of the University of Pittsburgh developed a scale to assess the comprehensive well-being of people in a university environment and named it the “Pitt Wellness Scale,” which evaluates participants in seven domains: physical, mental, social, financial, spiritual, occupational, and intellectual (Zhou and Parmanto, 2020). Essentially, Pitt Wellness Scale summarizes the main factors that influence well-being, which we localized with the permission of Dr. Leming Zhou.

As a country with a large population, China has a large proportion of university students in the world. According to United Nations Educational, Scientific, and Cultural Organization (UNESCO)^1^ statistics, the total number of people enrolled in higher education in the world was 220 million in 2016, and the number of people enrolled in higher education in China reached 43.886 million (UNESCO, 2019, 04-23). Therefore, it would be significant to validate the psychometric properties of the Pitt Wellness Scale in China, where more than one-fifth of the world’s higher education population is located. It can provide a measurement tool for assessing the population’s well-being in Chinese university environment. To the best of our knowledge, we are the only ones who have localized it so far, and the results we obtained can provide a reference for future localization in other countries.

### Aims and Expected Results

Due to the differences in cultural and educational backgrounds between the United States and China, there should be some differences in the dimensional aspects of the scale. In this study, we examine the psychometric properties of the Pitt Wellness Scale in a sample of Chinese university environments, comparing the differences between the findings and the original scale and exploring the specific reasons for them.

On the other hand, we estimated that the well-being of people in the university environment is related to the role in the university, gender, whether they are an only child, ethnic groups, education background, subject, household income, monthly living expenses, and grade, so we compared individual well-being scores with general information to validate our view.

## Materials and Methods

### Translation and Cultural Adaptation

We obtained permission from Dr. Leming Zhou before translating and validating the Pitt Wellness Scale and followed the systematic process of Brislin translation (Brislin, 1970). Two graduate students independently translated the Pitt Wellness Scale into Chinese, and together with the researchers, they compared the contents of the translated Chinese version of the scale, discussed and corrected the differences in the translation of the scale, and finally obtained the first draft of the Chinese version. We had two English experts without exposure to the scale translate the first draft of the Chinese back into English, following Brislin’s translation-reverse translation method (Brislin, 1970). Finally, the expert group compared and discussed the original scale, the first draft of the Chinese translation, and the back-translated English scale. The controversial items were revised, focusing on language and cultural adjustments to make the content of the scale more in line with the actual situation in China. The original scale had 44 items and seven dimensions, eight of which were self-assessment levels for each domain and overall wellness, and these eight items experts recommended deleting. Because the scale involves some financial domain and job-related issues, Chinese college students are unlikely to be exposed to work during university, and they will have stable jobs after graduation and entering society. Therefore, combined with the actual situation in China and the sample population (faculty, staff, and students) in the university environment, experts suggest modifying some items. The adapted items measure the student’s approximate grasp of the future occupation they will pursue and predict the individual’s competence. The specific modifications are shown in Table 1.

**TABLE 1:** Modification items of Chinese Revision of the Pitt Wellness Scale.

Pitt Wellness Scale	Chinese Revision of the Pitt Wellness Scale
(1) My income is adequate for my current needs. (2) I feel I have input on deciding how my job gets done.	(1) My income/living expenses is adequate for my current needs. (2) I feel I have input on deciding how my job/future job gets done.
(3) I am satisfied with the amount of time required by my job duties.	(3) I am/will be satisfied with the amount of time required by my job/future job duties.
(4) My employer provides me many career development opportunities.	(4) My employers, leaders, teachers, or universities provide me many career development opportunities.
(5) I feel comfortable working with my colleagues.	(5) I feel/will feel comfortable working with my colleagues/future colleagues.
(6) My work and life are well-balanced.	(6) My work/future occupation and life are/will be well-balanced.
(7) My job security is high (8) I am satisfied with the quality of my work.	(7) I think my job/future job security is high. (8) I am satisfied with the quality of my work/study.

### Design and Study Population

A cross-sectional survey was carried out in Liaoning Province (Northeast China) and Shandong Province (Eastern China) from April to May 2021. Participants were college faculty, staff, and students of five universities (Jinzhou Medical University, Shandong Medical College, BOHAI University, Jinzhou Normal College, Panjin Vocational and Technical College). We chose a professional platform called “wenjuanxing,” a data collection questionnaire that is the functional equivalent of Amazon’s Mechanical Turk platform. A total of 1870 participants took the survey, and after examining each data, participants who did not complete the scale and had obvious logical errors (student’s educational background was inconsistent with his grade) were excluded. The survey was anonymous except 50 participants were required to write their student numbers or job number as the test–retest participants. Four weeks later, 50 participants who joined the first test were recruited to evaluate the test–retest reliability. All participants were native Mandarin speakers and provided informed consent before participating in the study. The study procedures followed the ethical standards of the Ethics Committee of Jinzhou Medical University (JZMULL2021009) and the 1964 Declaration of Helsinki and subsequent amendments.

### Materials

#### Pitt Wellness Scale

Pitt Wellness Scale, developed by Dr. Zhou and Parmanto of the University of Pittsburgh (Zhou and Parmanto, 2020), is used to evaluate people’s comprehensive well-being in the university environment, including faculty, staff, and students. Pitt Wellness Scale consists of 44 items and seven dimensions, namely physical domain, mental domain, social domain, financial domain, spiritual domain, occupational domain, and intellectual domain, and the Cronbach alpha coefficient was 0.933. For most of the items, response options ranged on a scale from 1 (strongly agree) to 7 (strongly disagree). Eight items (deleted from the Chinese version) were selected on the scale from 1 (excellent) to 5 (terrible). The options of the pain item ranged from 0 (no pain) to 10 (most severe pain ever). On this scale, the higher the total score, the lower the well-being. The translation of the Pitt Wellness Scale to the Chinese Revision of the Pitt Wellness Scale has been discussed earlier.

#### General Well-Being Schedule

The General Well-Being Schedule (GWBS) is a standardized test tool developed by the National Center for Health Statistics in 1977 to evaluate participants’ well-being (Fazio, 1977). The internal consistency reliability coefficient of GWBS was 0.91 for males and 0.95 for females. This study used a revised version by Chinese scholar Jianhua Duan, which uses the first 18 items of the scale to evaluate participants’ general well-being. The scale consists of six dimensions, and the higher the score, the stronger the subjective well-being.

### Data Analysis

#### Validity Analysis

##### Construct Validity

All statistical analyses of the data were performed using IBM SPSS version 25.0 and AMOS version 24.0 (IBM Corporation, New York, NY, United States). The construct validity of the Chinese Revision of the Pitt Wellness Scale was tested by exploratory factor analysis (EFA) and validation factor analysis (CFA). We randomly divided the data into two groups. One group consisted of 958 individuals for EFA, and 912 individuals for CFA. The Kaiser–Meyer–Olkin (KMO) statistic and Bartlett test of sphericity (BTS) test were used to assess the factor ability of the correlation matrix in sample 1 used for EFA (Bartlett, 1954; Kaiser and Cerny, 1979). Data were considered suitable for factor analysis when the KMO was greater than 0.60 and Bartlett test of sphericity test was significant (*P* < 0.05).

Sample 2 was subjected to the CFA test with a test level of α = 0.05. We used the following absolute and incremental fit metrics to assess the fit of the structural model: (1) chi-square degree of freedom (χ^2^/df, less than 5 is reasonable value and the model is acceptable); (2) root mean square error of approximation (RMSEA, 0.08 as a cutoff value for poorly fitted models); (3) standardized root mean square residual (SRMR) with values less than 0.08 are generally considered a good fit; (4) comparative fit index (CFI), the value range is 0.0 –1.0, the closer to 1.0 means a good fit (CFI ≥ 0.90); and (5) Tucker Lewis Index (TLI), which range is also between 0.0 – 1.0, and TLI ≥ 0.9 indicates a good fit (Wheaton et al., 1977; Hooper et al., 2008).

##### Discriminant Validity

Discriminant validity was determined by ranking the total scores of the Chinese Revised of the Pitt wellness Scale from highest to lowest, assigning the 27% with the highest scores to the high group and the 27% with the lowest scores to the low group, and analyzing the scores of each item in the two groups using a two-tailed independent samples *t*-test. Discriminant validity was considered good if the scores of each item in the two groups reached a significant level (*p* < 0.05).

##### Content Validity

The content validity of the Chinese Revision of the Pitt Wellness Scale was evaluated using the expert assessment method and content validity index (CVI). Experts assessed the relevance of each item to the corresponding dimension. The CVI consists of two components, the item-level content validity index (I-CVI) and the Average of the I-CVIs for all items on the scale (S-CVI/Ave) (Lynn, 1986). I-CVI must be above 0.78, and S-CVI/Ave should be above 0.90 (Polit and Beck, 2006).

##### Criterion Validity

This study used the correlation method for criterion analysis, and the GWBS as the criterion instrument to make preliminary inferences about the validity of the Chinese Revision of the Pitt Wellness Scale.

#### Reliability Analysis

Reliability for internal consistency of the scale was examined using the Omega coefficient, and a value of 0.8 or better indicates good internal consistency (Taylor, 2021). In addition, we measured the Cronbach alpha coefficient, split-half reliability, and reliability between different samples of males and females. The scale’s stability is reflected by the retest correlation coefficient (intraclass correlation coefficient, ICC), namely retest reliability.

##### Differential Analysis of Socio-Demographic Information

Differences between well-being and socio-demographic information classification were tested by independent samples *t*-test or single-factor ANOVA. Bonferroni’s test was used to calibrate test levels for pairwise comparisons.

**FIGURE 1:**
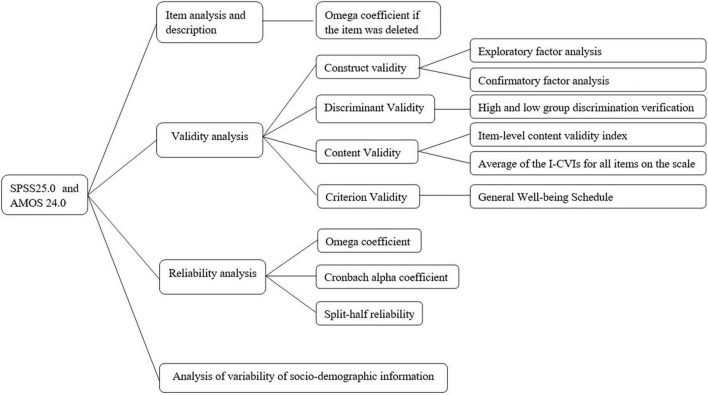
China Revision of the Pitt Wellness Scale data analysis process.

The data analysis and validation process are shown in Figure 1.

## Results

### Descriptive Statistics

We surveyed 1870 university faculty, staff, and students. Considering that the university environment is overwhelmingly student-based, students are the mainstay in our study (1791/1870, 95.7%), and only a few participants were faculty (70/1870, 3.7%) and staff (9/1870, 0.5%). Most participants were female (1494/1870, 79.9%), with a mean age of 20.61 years (SD 6.123, age range: 16–58 years), and more than three-quarters of the research participants majored in medicine (1428/1870, 76.35%). Further details on demographics are provided in Table 2. The mean (SD) scores of participants for each item of the Chinese Revision of the Pitt Wellness Scale are shown in Table 3.

**TABLE 2:** Participants’ characteristics (*n* = 1870).

Characteristic	*n*	%
**Role**		
Student	1791	95.8
Faculty	70	3.7
Staff	9	0.5
**Gender**		
Male	376	20.1
Female	1494	79.9
**Only Children**		
Yes	766	40.9
No	1104	59.0
**Ethnic groups**		
Han nationality	1515	81.0
Ethnic minority	355	19.0
**Education background**		
Junior college or below	1239	66.2
Bachelor’s degree	554	29.6
Master’s degree	66	3.5
Doctoral degree or above	11 0.6	
**Marital status**		
Single	1793	95.9
Married	73	3.9
Divorced	4	0.2
**Major**		
Medicine	1428	76.3
Education	352	18.8
Engineering	26	1.4
Management	7	0.4
Science	37	2.0
Agronomy	10	0.5
Philosophy	10	0.5
**Household income**		
≤CNY5000	934	49.9
CNY5001—10000 (2)	718	38.4
CNY10001—20000 (3)	158	8.4
>CNY20000 (4)	60	3.2
**Monthly living expenses**		
**(students)**		
≤CNY1000 (1)	448	23.9
CNY1001—1500 (2)	920	49.2
CNY1501—2000 (3)	333	17.8
>CNY2000	90	4.8
**Grade(students)**		
Freshman	1336	71.4
Sophomore	345	18.4
Junior	63	3.4
Senior	9	0.5
First-year postgraduate	38	2.1
**School**		
Jinzhou Medical University	669	35.8
Shandong Medical College	306	16.4
BOHAI University	123	6.6
Jinzhou Normal College	250	13.4
Panjin Vocational and Technical College	522	27.9

**TABLE 3:** Mean (*SD*) scores of participants for each item of the Chinese Revision of the Pitt Wellness Scale (*N* = 1870).

Items on Chinese Revision of the Pitt Wellness Scale	Mean (*SD*)
(1) I feel rested when I wake up in the morning.	4.01 (1.90)
(2) Each week, I exercise moderately for at least 30 min (for instance, walking briskly, bicycling slower than 10 miles per hour, playing tennis, and ballroom dancing).	3.25 (1.65)
(3) Because of my health status, I am physically able to exercise as much as I would like to.	3.02 (1.49)
(4) I usually have enough energy for everyday activities.	3.56 (1.62)
(5) My chronic pain level is (0 = no pain, 10 = most severe pain ever).	1.03 (1.91)
(6) My appetite has been good recently.	3.01 (1.52)
(7) I am generally satisfied with my quality of life.	2.85 (1.31)
(8) I am generally self-accepting.	2.66 (1.24)
(9) I feel hopeful about the future.	2.46 (1.27)
(10) I feel that I have control over my emotions.	2.66 (1.24)
(11) I believe that life is what you make it.	2.29 (1.17)
(12) I am open to new opportunities if my first plan does not work out.	2.27 (1.14)
(13) I am living in a safe community.	2.07 (1.09)
(14) When something good happens to me, I share the experience with my family and/or friends.	1.99 (1.10)
(15) I am satisfied with my ability to meet the needs of people who depend on me.	2.44 (1.20)
(16) I am satisfied with my current level of social activities.	2.66 (1.33)
(17) I have people in my life who care about me.	2.00 (1.10)
(18) If I incur an unexpected above average expense, I would still be stable financially.	3.25 (1.53)
(19) I have someone to help with my financial affairs, if needed.	3.38 (1.62)
(20) I am saving for retirement and for emergencies.	4.47 (1.89)
(21) My income/living expenses is adequate for my current needs.	2.90 (1.50)
(22) I feel that my life is meaningful.	2.32 (1.23)
(23) I feel inner and/or spiritual strength in difficult times.	2.59 (1.25)
(24) I have a sense of direction for my life.	2.63 (1.27)
(25) I know what is really important in my life.	2.44 (1.24)
(26) My personal beliefs (religious or not) help me to cope with difficulties in life.	3.13 (1.57)
(27) I feel I have input on deciding how my job/future job gets done.	2.52 (1.20)
(28) I am/will be satisfied with the amount of time required by my job/future job duties.	2.78 (1.32)
(29) My employers, leaders, teachers, or universities provide me many career development opportunities.	2.97 (1.35)
(30) I feel/will feel comfortable working with my colleagues/future colleagues.	2.59 (1.19)
(31) My work/future occupation and life are/will be well-balanced.	2.73 (1.23)
(32) I think my job/future job security is high.	2.76 (1.27)
(33) I am satisfied with the quality of my work/study.	2.86 (1.32)
(34) I am aware of my intellectual strengths.	2.76 (1.26)
(35) I can rely upon my talents and skills to handle unexpected situations.	2.83 (1.28)
(36) I am satisfied with my ability to make decisions.	2.85 (1.28)

### Item Analyze

We analyzed the items on the scale (36 items). The reliability analysis showed that the overall Omega coefficient was 0.962 (95% CI: 0.959–0.964), but the internal consistency of the scale would be improved if items 5, and 20 were deleted, as detailed in Table 4. We initially conducted EFA with no factor loadings for item 5. When experts assessed the content validity, the I-CVI for the 20th item was 0.429. So, items 5 and 20 were deleted after expert deliberation.

**TABLE 4:** Omega coefficient if the item is deleted (*N* = 1870).

Item	Omega coefficient if the item was deleted
1	0.962
2	0.962
3	0.961
4	0.961
**5**	**0.964**
6	0.960
7	0.960
8	0.960
9	0.960
10	0.960
11	0.960
12	0.960
13	0.961
14	0.960
15	0.960
16	0.961
17	0.961
18	0.961
19	0.961
**20**	**0.963**
21	0.961
22	0.960
23	0.960
24	0.960
25	0.960
26	0.961
27	0.960
28	0.960
29	0.960
30	0.960
31	0.960
32	0.960
33	0.960
34	0.960
35	0.960
36	0.960

*Bold are deleted items.*

### Validity Analysis

#### Construct Validity

**FIGURE 2:**
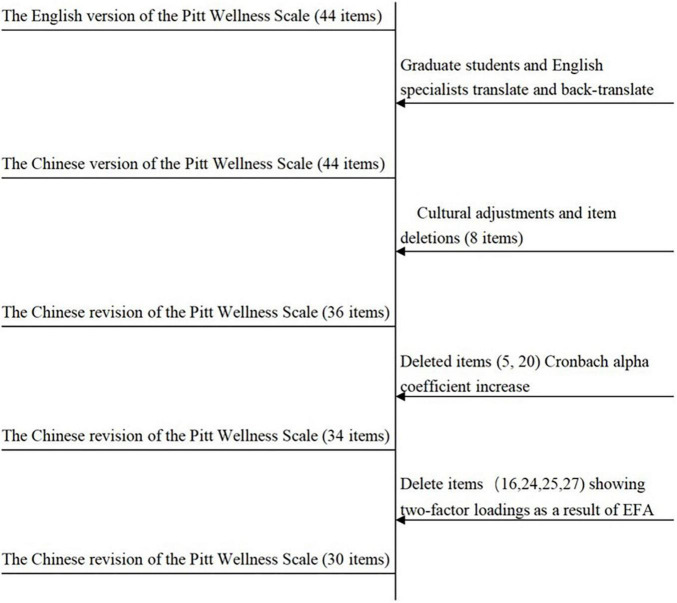
China Revision of the Pitt Wellness Scale item deletion flow chart.

We first checked the factorizability of the 34-items matrix in Sample 1 (*n* = 958). The Bartlett test of sphericity was statistically significant (χ^2^_958_ = 27835.939; *P* < 0.001), and the KMO index was 0.971, which is above the lowest value of 0.6. The results indicate a sufficient correlation between the variables, and the matrix is suitable for factor extraction. Principal axis factoring (PAF) was used to determine the number of possible factors and retain results with rotated factor loadings greater than 0.40. The results showed that the factor loadings of most of the question items were on a single factor, while the factor loadings of 16, 24, 25, and 27 items appeared on two different factors, which were deleted after expert discussion (Boone et al., 1998). See Supplementary Appendix 1 for details. The specific deletion process of Pitt Wellness Scale items is shown in Figure 2. After deleting the items, we performed EFA again on the remaining 30 items. The results showed the Bartlett test of sphericity was statistically significant (χ^2^_958_ = 23108.158; *P* < 0.001), and the KMO index was 0.966. The PAF yielded five common factors with eigenvalues > 1 (orthogonally rotated by the maximum variance method), explaining 64.321% of the total variance, which was different from the 7-factor theoretical construct model of the original scale. The results of the factor loadings are shown in Table 5. The correlations among factors in the Chinese Revision of the Pitt Wellness Scale are shown in Table 6.

**TABLE 5:** Rotated factor loadings of the exploratory factor analysis with 30 items (*N* = 958).

Items	Factor1	Factor2	Factor3	Factor4	Factor5
1	0.652				
2	0.681				
3	0.689				
4	0.797				
6	0.540				
7		0.504			
8		0.674			
9		0.713			
10		0.622			
11		0.663			
12		0.628			
13			0.613		
14			0.726		
15			0.575		
17			0.687		
18				0.675	
19				0.595	
21				0.608	
22		0.646			
23		0.632			
26		0.411			
28					0.640
29					0.629
30					0.602
31					0.753
32					0.701
33					0.810
34					0.793
35					0.792
36					0.766

**TABLE 6:** Correlations among factors in the Chinese Revision of the Pitt Wellness Scale (*N* = 1870).

Factor	Factor1	Factor2	Factor3	Factor4	Factor5	Number of items
Factor1	—	—	—	—	—	5
Factor2	0.551**	—	—	—	—	9
Factor3	0.422**	0.728**	—	—	—	4
Factor4	0.460**	0.569**	0.500**	—	—	3
Factor5	0.500**	0.752**	0.650**	0.574**	—	9
Total-score	0.703**	0.915**	0.783**	0.708**	0.892**	30

**P < 0.01. —Not available.

**FIGURE 3:**
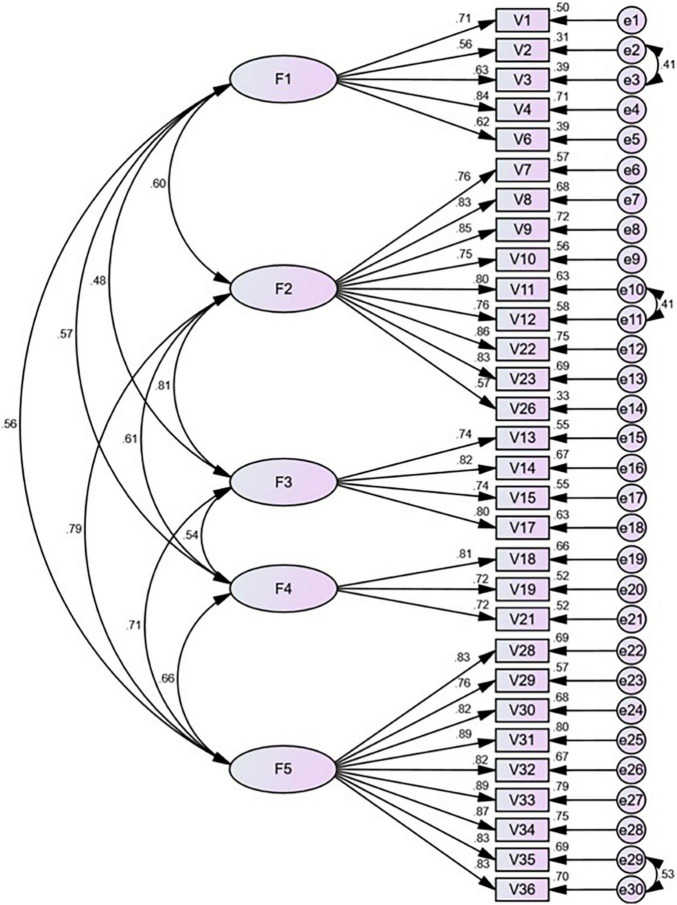
Standardized five-factor structural model for the China Revision of the Pitt Wellness Scale (*n* = 906, 30 items). F1 (Physical domain), F2 (Mental and Spiritual domain), F3 (Social domain), F4 (Financial domain), F5 (Occupational and intellectual domain) (We named Factor 2 as the psychological domain and Factor 5 as the competent domain. See the discussion section for more details).

Confirmatory factor analysis was performed on sample 2 (*n* = 912). The five-factor structural model fitting indexes met the fitness criteria, and the results are shown in Table 7. All indicators were basically superior to the original model index (Zhou and Parmanto, 2020). The standardized path analysis is shown in Figure 3.

**TABLE 7:** Indicator fit of the five-factor structural model of the Chinese Revision of the Pitt Wellness Scale (*n* = 912).

Model	χ^2^	df	χ^2^/df	CFI	TLI	SRMR	RMSEA [90% CI]
5-factor model	1796.460	392	4.583	0.933	0.926	0.0455	0.063 [0.060–0.066]

*χ^2^, chi-square; df, degrees of freedom; CFI, comparative fit index; TLI, Tucker–Lewis index; SRMR, standardized root mean residual; RMSEA, root mean square error of approximation; 90% CI, 90% confidence interval.*

#### Discriminant Validity

The total scores of the Chinese Revision of the Pitt Wellness Scale were ranked from highest to lowest, with the top 27% being the high group and the bottom 27% being the low group. In this study, critical value scores were 66 and 102, respectively. The results indicated that the scores of each item were statistically significant (*P* < 0.05) in both the high and low partitions, with good discriminatory properties that could effectively measure the degree of response of different participants. Results are shown in Table 8.

**TABLE 8:** Score comparison between high-score and low-score groups (*N* = 1870).

Items	Low-score group, mean (*SD*)	High-score group, mean (*SD*)	*P*-value
1	2.63(1.74)	5.02(1.46)	<0.001
2	2.10(1.26)	4.19(1.55)	<0.001
3	1.97(1.12)	3.96(1.41)	<0.001
4	2.22(1.33)	4.66(1.29)	<0.001
6	1.91(1.08)	4.02(1.43)	<0.001
7	1.70(0.87)	4.12(1.03)	<0.001
8	1.56(0.69)	3.92(0.96)	<0.001
9	1.39(0.63)	3.85(0.99)	<0.001
10	1.62(0.79)	3.78(1.05)	<0.001
11	1.37(0.54)	3.50(1.04)	<0.001
12	1.40(0.57)	3.42(1.07)	<0.001
13	1.28(0.49)	3.07(1.11)	<0.001
14	1.24(0.49)	2.99(1.19)	<0.001
15	1.42(0.65)	3.49(1.01)	<0.001
17	1.26(0.48)	3.00(1.15)	<0.001
18	1.95(1.18)	4.33(1.18)	<0.001
19	2.14(1.40)	4.38(1.20)	<0.001
21	1.74(0.99)	3.98(1.28)	<0.001
22	1.28(0.51)	3.68(0.99)	<0.001
23	1.49(0.70)	3.88(0.93)	<0.001
26	1.83(1.23)	4.30(1.08)	<0.001
28	1.54(0.73)	4.06(0.93)	<0.001
29	1.71(0.89)	4.15(0.98)	<0.001
30	1.50(0.68)	3.79(0.90)	<0.001
31	1.51(0.66)	3.97(0.85)	<0.001
32	1.55(0.72)	3.97(0.88)	<0.001
33	1.59(0.80)	4.11(0.90)	<0.001
34	1.56(0.76)	3.99(0.86)	<0.001
35	1.60(0.79)	4.01(0.84)	<0.001
36	1.63(0.82)	4.03(0.90)	<0.001

#### Content Validity

The content validity of the Chinese Revision of the Pitt Wellness Scale was assessed using the expert assessment method (Hambleton et al., 1978). The expert group consists of ten people, including three psychology experts, four medical experts proficient in Chinese and English, and three professors of education. The results of content validity analysis showed that the 20th item (I am saving for retirement and for emergencies) had a low I- CVI of 0.600, which indicated that the 20th item was indeed not suitable for the Chinese population, and was consistent with the results of our statistical analysis. The rest of the items ranged from 0.800 to 1, and the S-CVI/Ave was 0.975.

#### Criterion Validity

In this research, validity analysis was conducted using correlation analysis to make preliminary inferences about the validity of the Pitt Wellness Scale. The GWSB scale, which specifically measures well-being, was used as the validity instrument in this study. The correlation analysis between the GWSB and the Chinese Revision of the Pitt Wellness Scale showed a negative correlation and a statistically significant difference (*r* = –0.402, *p* < 0.001), indicating the Chinese Revision of the Pitt Wellness Scale can be used to assess comprehensive well-being for people in university environment.

### Reliability Analysis

Reliability analysis results indicated that the Chinese Revision of the Pitt Wellness Scale (30 items) has perfect internal consistency, with the overall Omega coefficient being 0.958 (95% CI: 0.955–0.961), which was higher than the overall Cronbach alpha coefficient of 0.933 for the original scale (44 items) (Zhou and Parmanto, 2020). Omega coefficients for the five factors are 0.820, 0.933, 0.868, 0.794, and 0.954, all of which greater than the minimum acceptable value of 0.7. In addition, the Cronbach alpha coefficient was 0.959 and the split-half reliability was 0.890, both showing good reliability. We did reliability tests separately for gender, and the results also showed satisfactory reliability, as detailed in Table 9.

**TABLE 9:** Omega coefficients and Cronbach alpha coefficient of participants.

Classify	Omega coefficient	Cronbach alpha coefficient
Males (*N* = 376)	0.968	0.968
Females (*N* = 1494)	0.955	0.957
The total sample (*N* = 1870)	0.958	0.959

After 4 weeks, 50 participants were retested. The test-retest reliability was 0.821, and the correlation coefficient was greater than 0.7, indicating that the Chinese Revision of the Pitt Wellness Scale has good retest reliability.

### Differential Analysis of Socio-Demographic Information

There were no significant differences in the Chinese Revision of the Pitt Wellness Scale on whether one is an only child, ethnic groups, or grade. However, there were statistically significant differences in the role of university, gender, education background, major, household income, and monthly living expenses. The specific results are shown in Table 10. The effects of socio-demographic variables on well-being in each domain are shown in Table 11. The comparison of the means and differences of well-being in various domains for different socio-demographic variables are shown in Supplementary Appendix 2.

**TABLE 10:** Comparison of the Chinese Revision of the Pitt Wellness Scale of participants with different characteristics.

Characteristic	Mean (*SD*)	*P*-value	Pairwise differences^a^
**Role**		**0.002**	
Student	83.67(27.37)		
Faculty and Staff	73.76(29.22)		
**Gender**		**0.001**	
Male	78.78(29.68)		
Female	84.38(26.83)		
**Only Children**		0.649	
Yes	82.88(28.54)		
No	83.51(26.79)		
**Ethnic groups**		0.067	
Han nationality	83.80(27.38)		
Ethnic minority	80.91(27.97)		
**Education background**		**0.030**	
Junior college or below (1)	84.21(27.48)		
Bachelor’s degree (2)	82.25(27.18)		**(3)<(1), (2)**
Master’s degree (3)	74.91(30.39)		
Doctoral degree or above (4)	76.55(20.72)		
**Subject**		**0.014**	
Medicine	84.27(25.71)		
Non-medical	80.30(32.64)		
**Household income**		**<0.001**	
≤ CNY5000 (1)	86.01(26.98)		
CNY5001—10000 (2)	82.14(27.19)		**(1)>(2), (3), (4)**
CNY10001—20000 (3)	76.70(28.92)		**(2)>(3), (4)**
>CNY20000 (4)	70.98(29.24)		
**Monthly living expenses** **(students)**		**0.005**	
≤ CNY1000 (1)	84.38(27.04)		
CNY1001—1500 (2)	85.17(26.56)		**(1)>(3), (4)**
CNY1501—2000 (3)	80.19(28.08)		**(2)>(3), (4)**
>CNY2000 (4)	77.78(32.56)		
**Grade(students)**		0.058	
Freshman	83.49(26.64)		
Sophomore	85.97(27.82)		
Junior	75.48(36.94)		
Senior	87.67(30.27)		
First-year postgraduate	79.51(27.66)		

*Bold values correspond to statistically significant correlations (p < 0.05).*

*^a^Pairwise differences were p < 0.05 (Bonferroni corrected).*

**TABLE 11:** Effect of socio-demographic variables on well-being in each domain (*N* = 1870).

Domain and characteristic	*P*-value
**Physical domain**	
Role	**<0.001**
Gender	**<0.001**
Only Children	0.114
Ethnic groups	0.343
Education background	**<0.001**
Subject	**0.009**
Household income	**0.041**
Monthly living expenses	0.402
Grade	**<0.001**
**Psychological domain**	
Role	0.092
Gender	0.057
Only Children	0.074
Ethnic groups	**0.017**
Education background	0.197
Subject	0.330
Household income	**0.001**
Monthly living expenses	**0.014**
Grade	0.230
**Social domain**	
ROLE	0.465
Gender	0.898
Only Children	0.432
Ethnic groups	0.216
Education background	0.778
Subject	0.207
Household income	**0.013**
Monthly living expenses	0.080
Grade	0.195
**Financial domain**	
ROLE	**0.004**
Gender	**<0.001**
Only Children	**0.007**
Ethnic groups	0.999
Education background	**<0.001**
Subject	**<0.001**
Household income	**<0.001**
Monthly living expenses	**<0.001**
Grade	0.053
**Competent domain**	
Role	**0.044**
Gender	0.066
Only Children	0.245
Ethnic groups	**0.004**
Education background	**<0.001**
Subject	**0.004**
Household income	**<0.001**
Monthly living expenses	**<0.001**
Grade	0.053

*Bold values correspond to statistically significant correlations (p < 0.05).*

### Distribution of Participants’ Well-Being Degree

To assess the comprehensive well-being degree for people in the University environment, a total score was calculated for each participant and transformed into a *Z*-score in the sample (*N* = 1870) (Gao et al., 2020). Apply the *Z*-score assessment criteria as follows: (1) Subjects were considered wellness if the *z*-score was below –1; (2) A *Z*-score between –1 and 0 (mean value) was considered the subject to be low unwellness; (3) A *Z*-score between 0 and 1 was be regarded the subject to be moderately unwellness; (4) A *Z*-score greater than 1 but less than 2 was considered the subject to be severe unwellness; (5) Subjects with a *Z*-score above 2 were considered very severe unwellness. The statistical results showed that 15.99% (299/1870), 36.20% (677/1870), 29.84% (558/1870), 15.72% (294/1870), and 2.25% (42/1870) were in the no, low, moderate, severe, and very severe unwellness groups, respectively.

## Discussion

### Chinese Revision of the Pitt Wellness Scale

In this study, the Pitt Wellness Scale developed by Leming Zhou (Zhou and Parmanto, 2020) was translated into Chinese with some adaptations, and its psychometric properties were tested in a large number of Chinese university faculty, staff, and students to investigate whether the Chinese Revision of the Pitt Wellness Scale could be applied in Chinese university environment. The results of the analysis showed that the Chinese revision of the scale has good reliability and validity.

The scale currently used to assess well-being in China is the GWBS. In our study the GWBS is not only a standard instrument but also the reliability of the scale was compared with the Chinese Revision of the Pitt Wellness Scale, as shown in Table 9, the reliability of the Chinese Revision of the Pitt Wellness Scale is better than the GWBS and can be applied to the university environment in the population.

In contrast to the seven-factor theoretical construct model of the original English scale, our findings support a five-factor structure consisting of 30 items. Eight items from the original scale (self-assessment of each domain and overall health level) were recommended for deletion after expert deliberation. The seven-factor in the original scale was named physical domain, mental domain, social domain, financial domain, spiritual domain, occupational domain, and intellectual domain. However, as shown in Table 5, the mental domain and spirit domain were on the same dimension in our study, and the same occurred for the occupational domain and intellectual domain. We consider that there are two reasons for the difference in structure from the original scale: first, in the translation process, we made a few adjustments to the items that did not fit the actual situation in China, which affected the structure of the original scale to some extent; second, the difference may be due to the sample population. In the original scale, the sample consisted mainly of faculty and staff, with a small proportion of students. In contrast, students made up a large percentage of the sample in our study because they are a major part of the population in the university environment. We investigated the people’s well-being at multiple universities with a large sample, while the original scale surveyed the well-being of the population at the University of Pittsburgh with a small sample compared to ours.

We modified some items in the financial, occupational, and intellectual domains according to the actual situation of Chinese universities. A study of financial differences between Chinese and American college students showed that American college students were more financially confident than Chinese, and Chinese students reported lower levels of financial well-being (Norvilitis and Mao, 2013). Our study is consistent with this, except for our revised item (item 21 in Table 3), the average of Chinese participants in the financial domain (18, 19 items in Table 3) is higher than the sample in the original scale (Zhou and Parmanto, 2020). This result is primarily due to cultural and educational differences between China and America. Certain work experiences and parental education can influence children’s financial attitudes (Shim et al., 2010). A survey in China found that 11% of Chinese parents paid for their children’s credit card debt while they were still in their twenties, and students are supported by their parents during their college years (Norvilitis and Mao, 2013). However, most American students pay for their education through work or government loans and repay these expenses themselves (Baum et al., 2013; Walsemann et al., 2015). In China, students have little exposure to work before attending university, and university planning place too much emphasis on teaching, with few training programs mentioning work experience, and internships only taking place in the final year of graduation. Accordingly, taking into account the student population in the university environment, the term “living expenses” was added to the item “My income is adequate for my current needs” in the Chinese Revision of the Pitt Wellness Scale, and some modifications were made to the occupational domain items. We revised item 33 (in Table 3), “I am satisfied with the quality of my work” to “I am satisfied with the quality of my work/study.” Since this item is related to work, it was suggested by the expert group during the discussion that it should be categorized as occupational domain, but in the original scale, it was classified as intellectual domain, which is one of the reasons why the dimensional division is different from the original scale.

In terms of content, there are relatively reasonable explanations for the changes in these dimensions. In particular, the items in the mental domain (items 7–12 in Table 3) and the spiritual domain (items 22, 23, 26 in Table 3) of the original scale express a sense of the meaning of life and predict life satisfaction. RUFF and Singer interpreted psychological well-being as the result of leading a happy life (Ryff and Singer, 1998). The interpretation of psychological well-being is not a measure of well-being *per se*, but an indicator that a person is living a good life (Ryan et al., 2008). In the Oxford English Dictionary, psychology has both mental and spiritual meaning. In addition, there is a close connection between the occupational domain (items 28–32 in Table 3) and the intellectual domain (items 33–36 in Table 3). It is well known that occupation is highly correlated with intelligence (Matazaro, 1972; Leli and Filskov, 1979). Academic and occupational achievement can be enhanced by good intelligence (Lowenstein et al., 1983). Intelligence is the basis of occupation, and occupation is the external expression of intelligence, which we collectively refer to it as competence. The remaining three factors were consistent with the analysis results of the original scale and were named as physical domain, social domain, and financial domain. According to the analysis of the items’ specific content and factor connotations, and after consideration by the expert group, the mental and spiritual domains were summarized as psychological domain, and the occupational and intellectual domains were summarized as competent domain, which is more in line with the Chinese cultural context and research findings.

### Differences in Well-Being Between Different Role

The results show that the mean scores of students (83.67 ± 27.37) are higher than those of faculty and staff (73.76 ± 29.22), and the difference is statistically significant (*p* < 0.05), that is, faculty and staff have a higher sense of well-being. The reason we consider is that faculty and staff are economically free and have stable jobs, and their lives are stable. In contrast, college students have to face many uncertainties such as economics and personal competence, have less life experience (Hu and Kuh, 2003) than faculty and staff, and are less comfortable handling things than they are, thus reducing students’ sense of well-being.

### Differences in Well-Being Between Different Gender

In this study, females (84.38 ± 26.83) had lower well-being than males (78.78 ± 29.68), and the difference was statistically significant (*p* < 0.05). Males and females face different health risks due to different gender roles (Heise et al., 2019). The persistence of gender discrimination in employment and the responsibilities females have to bear in the family can cause them to be exposed to different diseases, disabilities and injuries (Messing et al., 2003; WHO, 2016). According to the Spanish results, females are more likely to report poor self-perceived health than males (Esteban-Gonzalo et al., 2021), consistent with our findings. Compared to the past, the concept of gender equality has been accepted by the public (Lewis, 2006). Females are more involved in social life and work (Hoffman, 1977). In the workplace, their requirements for themselves have become higher, and accordingly, they take on more responsibility and pressure (Belle, 1988). At the same time, from the perspective of personality, females are more emotionally sensitive and delicate than males, fluctuate easily, and pay too much attention to details (Oxford, 1993). Therefore, they are often troubled by some trivial matters in life, which reduces their well-being level.

### Differences in Well-Being Between Different Educational Backgrounds

From the perspective of educational level, there are significant differences (*p* < 0.05) in well-being among participants of different educational levels, which the well-being of participants with master’s degree or above are higher than that of participants with junior college and bachelor’s degree (see Table 9 for details). The more educated a person is, the more capable the individual is, the more peaceful and tolerant he or she is toward things, and the more happy he or she is compared to the average or less capable person (Dang, 2019; Weyns et al., 2021).

### Differences in Well-Being Between Different Majors

The study examined the variability of the subjects using the professional category (medical, non-medical) as a grouping variable and found significant differences (*p* < 0.05) in the professional category of the participants. Participants in the non-medical category (80.30 ± 32.64) had lower mean scores than those in the medical category (84.27 ± 25.71), indicating that subjects in the medical category had lower well-being. The professional category has a significant effect on the well-being of people in a university environment. Medical students are more stressed compared to other disciplines (General Medical Council, 2016). Many studies have shown that medical students around the world are more prone to depression, anxiety, and psychological distress than the general population, and that the incidence is relatively high among Chinese medical students in different specialties compared to medical students in other countries (Stewart et al., 1999; Dyrbye et al., 2006, 2008; Moir et al., 2018; Zeng et al., 2019). A meta-analysis by the National University of Singapore showed that the worldwide prevalence of depression in medical students was 28.0% (Puthran et al., 2016). Given this situation, medical universities should take appropriate and urgent interventions to reduce student stress and improve the well-being of their students.

### Differences in Well-Being Between Different Household Income

The results of this study show that the higher the household income, the stronger the well-being of participants, and the difference is statistically significant (*p* < 0.05) (see Table 9 for details). The monthly living expenses of students are positively correlated with household income, and the result is similar to that. The main predictor of poor subjective well-being was identified as household income in the study by Powell-Young (2012). We consider this due to the good economic level and living environment of families with household income, which can provide good material conditions. The participants of such families are more comfortable in daily consumption and have no economic burden (Taylor et al., 2017). However, participants with lower household incomes will face more pressure in the economy, life, and other stressors. Thus, participants with higher household incomes will have higher overall well-being than participants with lower household incomes.

### Differences in Well-Being in Terms of Only Children, Ethnic Groups, and Grades

Contrary to our expectations, we conducted independent *t*-tests of their well-being for whether they were only children, ethnic groups, and grades, and the results showed no statistical differences. To our surprise, there was no difference between whether one was an only child and well-being, which is different from the results of previous studies (Downey, 1995; Liu et al., 2010). We consider that it may be because parents love their children the same regardless of whether there are multiple children in the family or not, and they all make their children feel the same warmth. China has been treating all ethnic groups equally and has also introduced some policies to protect the rights and interests of minorities (Sautman, 2010; Zhou, 2021), such as extra points in high schools’ entrance exams and minority backbone programs. We did an ANOVA on the well-being of different grades, which did not show statistically significant differences. However, in terms of the mean well-being results, the well-being of senior students is lower. This may be due to the relatively high pressure of graduation and employment faced by seniors (Lim et al., 2018).

In China, there is no scale to measure the well-being of university students in such a comprehensive domain. Our current study extends the work to people in university environments. This paper discusses comprehensive well-being with general socio-demographic information. Further, more detailed individual domains of well-being with general socio-demographic information are not discussed. Detailed data are shown in Supplementary Appendix 2.

## Limitation

There are two major limitations to this study. First, we assess the well-being of people in the university environments in northern China, which is not a good representation of the entire country. Second, this study included a large number of students, but a relatively smaller number of faculty and staff. However, considering that the main population in the university environment is students, there is not much bias in our study, and it is more in line with the actual situation.

## Conclusion

The Pitt Wellness Scale measures the level of well-being of all people in university environment. The Chinese Revision of the Pitt Wellness Scale has 30 items, supports a five-factor structure, and the results have proven to be reliable. In this study, participants’ well-being was related to the role in the university, gender, major, educational background, household income, and living expenses. The scale should be applied to universities in different regions of China in future studies to explore the factors that influence the well-being of university populations for further interventions.

## Data Availability Statement

The original contributions presented in the study are included in the article/Supplementary Material, further inquiries can be directed to the corresponding author/s.

## Ethics Statement

The studies involving human participants were reviewed and approved by the Ethics Committee of Jinzhou Medical University. The patients/participants provided their written informed consent to participate in this study.

## Author Contributions

All the authors conceived the study. XY wrote the manuscript. CL designed and directed the study. YG, LW, YL, and SL collected the data for this study. HZ, HY, and HT performed the data analysis.

## Conflict of Interest

The authors declare that the research was conducted in the absence of any commercial or financial relationships that could be construed as a potential conflict of interest.

## Publisher’s Note

All claims expressed in this article are solely those of the authors and do not necessarily represent those of their affiliated organizations, or those of the publisher, the editors and the reviewers. Any product that may be evaluated in this article, or claim that may be made by its manufacturer, is not guaranteed or endorsed by the publisher.

## Footnotes

1
https://www.unesco.org/en

## References

[B1] American College Health Association (2017). *National College Health Assessment Fall 2019 Reference Group Data Report.* Hanover, MD: American College Health Association.

[B2] Bartlett M. S. (1954). A note on the multiplying factors for various χ2 approximations. *J. R. Statist. Soc. Ser. B* 1954 296–298. 10.1111/j.2517-6161.1954.tb00174.x

[B3] Baum S. Kurose C. McPherson M. (2013). An overview of American higher education. *Future Child.* 23 17–39. 10.1353/foc.2013.0008 25522644

[B4] Belle D. (1988). Gender differences in the social moderators of stress. *J. Appl. Psychol.* 70 258–274. 10.7312/mona92982-021

[B5] Boone K. B. Pontón M. O. Gorsuch R. L. González J. J. Miller B. L. (1998). Factor analysis of four measures of prefrontal lobe functioning. *Arch. Clin. Neuropsychol.* 13 585–595. 10.1016/s0887-6177(97)00074-7 14590619

[B6] Brislin R. W. (1970). Back-Translation for Cross-Cultural Research. *J. Cross Cult. Psychol.* 1 185–216. 10.1037/a0021453 21038953

[B7] Chan D. W. (1998). Stress, coping strategies, and psychological distress among secondary school teachers in Hong Kong. *Am. Educ. Res. J.* 35 145–163. 10.3102/00028312035001145

[B8] Chan D. W. Hui E. K. (1995). Burnout and coping among Chinese secondary school teachers in Hong Kong. *Br. J. Educ. Psychol.* 65 15–25. 10.1111/j.2044-8279.1995.tb01128.x 7727264

[B9] Cools C. I. de Vries N. M. Bloem B. R. (2020). Happiness: a novel outcome in parkinson studies? *J. Parkinsons Dis.* 10 1261–1266. 10.3233/jpd-201999 32568107 PMC7458517

[B10] Dang T. (2019). “Subjective Well-being at Old Ages: Does Educational Background Matter?,” in *Paper Presented at the The Asian Conference on the Social Sciences 2019* (Nagoya: The International Academic Forum).

[B11] Diener E. Seligman M. E. (2004). Beyond money: toward an economy of well-being. *Psychol. Sci. Public Interest* 5 1–31. 10.1111/j.0963-7214.2004.00501001.x 26158992

[B12] Diener E. Emmons R. A. Larsen R. J. Griffin S. (1985). The satisfaction with life scale. *J. Personal. Assess.* 49 71–75. 10.1207/s15327752jpa4901_13 16367493

[B13] Downey D. B. (1995). When bigger is not better: family size, parental resources, and children’s educational performance. *Am. Sociol. Rev.* 1995 746–761. 10.2307/2096320

[B14] Dyrbye L. N. Thomas M. R. Shanafelt T. D. (2006). Systematic review of depression, anxiety, and other indicators of psychological distress among US and Canadian medical students. *Acad. Med.* 81 354–373. 10.1097/00001888-200604000-00009 16565188

[B15] Dyrbye L. N. Thomas M. R. Massie F. S. Power D. V. Eacker A. Harper W. (2008). Burnout and suicidal ideation among US medical students. *Ann. Internal Med.* 149 334–341. 18765703 10.7326/0003-4819-149-5-200809020-00008

[B16] Eckersley R. (2011). A new narrative of young people’s health and well-being. *J. Youth Stud.* 14 627–638. 10.1080/13676261.2011.565043

[B17] Esteban-Gonzalo S. González-Pascual J. L. Gil-Del Sol M. Esteban-Gonzalo L. (2021). Exploring new tendencies of gender and health in university students. *Arch. Womens Ment. Health* 24 445–454. 10.1007/s00737-020-01087-z 33184725

[B18] Fazio A. F. (1977). A concurrent validational study of the NCHS General Well-Being Schedule. *Vital Health Stat.* 73 1–53. 10.1007/978-3-319-56782-2_1939-2 610049

[B19] Fung S. F. (2019). Psychometric evaluation of the Warwick-Edinburgh Mental Well-being Scale (WEMWBS) with Chinese University Students. *Health Qual. Life Outcomes* 17 1–9. 10.1186/s12955-019-1113-1 30871563 PMC6416978

[B20] Gao Y. Dai H. Jia G. Liang C. Tong T. Zhang Z. (2020). Translation of the Chinese version of the nomophobia questionnaire and its validation among college students: factor analysis. *JMIR Mhealth Uhealth.* 8:e13561. 10.2196/13561 32167480 PMC7101502

[B21] General Medical Council (2016). *Professional Behaviour and Fitness to Practice: Guidance for Medical Schools and Their Students: General Medical Council.* London: General Medical Council.

[B22] Govorova E. Benítez I. Muñiz J. (2020). How schools affect student well-being: a cross-cultural approach in 35 OECD countries. *Front. Psychol.* 11:431. 10.3389/fpsyg.2020.00431 32269538 PMC7109313

[B23] Hambleton R. K. Swaminathan H. Algina J. Coulson D. B. (1978). Criterion-referenced testing and measurement: a review of technical issues and developments. *Rev. Educat. Res.* 48 1–47. 10.3102/00346543048001001

[B24] Hartwell H. (2015). Mental health and wellbeing. *Perspect. Public Health* 135:35. 10.1177/1757913914561670 25568188

[B25] Heise L. Greene M. E. Opper N. Stavropoulou M. Harper C. Nascimento M. (2019). Gender inequality and restrictive gender norms: framing the challenges to health. *Lancet.* 393 2440–2454. 10.1016/S0140-6736(19)30652-X 31155275

[B26] Hills P. Argyle M. (1998). Positive moods derived from leisure and their relationship to happiness and personality. *Personal. Individ. Differ.* 25 523–535. 10.1016/s0191-8869(98)00082-8

[B27] Hoffman L. W. (1977). Changes in family roles, socialization, and sex differences. *Am. Psychol.* 32 644. 10.1037/0003-066x.32.8.644

[B28] Hooper D. Mullen J. Hooper D. Coughlan J. Mullen M. R. (2008). Structural equation modeling: guidelines for determining model fit. *Electr. J. Bus. Res. Methods* 6 53–60.

[B29] Hu S. Kuh G. D. (2003). Diversity experiences and college student learning and personal development. *J. College Stud. Dev.* 44 320–334. 10.1353/csd.2003.0026 34409987

[B30] Hui E. K. (1998). Guidance in Hong Kong schools: students’ and teachers’ beliefs. *Br. J. Guid. Counsel.* 26 435–448. 10.1080/03069889800760371

[B31] Kaiser H. F. Cerny B. A. (1979). Factor analysis of the image correlation matrix. *Educ. Psychol. Measure.* 39 711–714. 10.1177/001316447903900402

[B32] Leli D. A. Filskov S. B. (1979). Relationship of intelligence to education and occupation as signs of intellectual deterioration. *J. Consult. Clin. Psychol.* 47 702–707. 10.1037//0022-006x.47.4.702 500905

[B33] Lew B. Huen J. Yu P. Yuan L. Wang D. F. Ping F. (2019). Associations between depression, anxiety, stress, hopelessness, subjective well-being, coping styles and suicide in Chinese university students. *PLoS One* 14:e0217372. 10.1371/journal.pone.0217372 31260454 PMC6602174

[B34] Lewis J. (2006). Work/family reconciliation, equal opportunities and social policies: the interpretation of policy trajectories at the EU level and the meaning of gender equality. *J. Eur. Public Policy* 13 420–437. 10.1080/13501760600560490

[B35] Lim A. Y. Lee S. H. Jeon Y. Yoo R. Jung H. Y. (2018). Job-Seeking Stress, Mental Health Problems, and the Role of Perceived Social Support in University Graduates in Korea. *J. Kor. Med. Sci.* 33:e149. 10.3346/jkms.2018.33.e149 29736162 PMC5934522

[B36] Lindert J. Bain P. A. Kubzansky L. D. Stein C. (2015). Well-being measurement and the WHO health policy Health 2010: systematic review of measurement scales. *Eur. J. Public Health* 25 731–740. 10.1093/eurpub/cku193 25616594

[B37] Linton M.-J. Dieppe P. Medina-Lara A. (2016). Review of 99 self-report measures for assessing well-being in adults: exploring dimensions of well-being and developments over time. *BMJ Open* 6:e010641. 10.1136/bmjopen-2015-010641 27388349 PMC4947747

[B38] Liu R. X. Lin W. Chen Z. Y. (2010). School performance, peer association, psychological and behavioral adjustments: a comparison between Chinese adolescents with and without siblings. *J. Adolesc.* 33 411–417. 10.1016/j.adolescence.2009.07.007 19651433

[B39] Liu S. Onwuegbuzie A. J. (2012). Chinese teachers’ work stress and their turnover intention. *Int. J. Educat. Res.* 53 160–170. 10.1016/j.ijer.2012.03.006

[B40] Liu X. P. Zhou Y. Y. (2016). Research on the impact of job stress on job satisfaction of college teachers. *High. Educ. Explor.* 153 125–129.

[B41] Lowenstein L. F. Meza M. Thorne P. E. (1983). A study in the relationship between emotional stability, intellectual ability, academic attainment, personal contentment and vocational aspirations. *Acta Psychiatr. Scand.* 67 13–20. 10.1111/j.1600-0447.1983.tb00326.x 6846034

[B42] Lynn M. R. (1986). Determination and quantification of content validity. *Nurs. Res.* 1986:17. 10.1097/00006199-198611000-00017 3640358

[B43] Matazaro J. (1972). *Wechsler’s Measurement and Appraisal of Adult Intelligence.* Oxford: Oxford University Press Inc, 209.

[B44] Messing K. Punnett L. Bond M. Alexanderson K. Pyle J. Zahm S. (2003). Be the fairest of them all: challenges and recommendations for the treatment of gender in occupational health research. *Am. J. Ind. Med.* 43 618–629. 10.1002/ajim.10225 12768612

[B45] Moir F. Yielder J. Sanson J. Chen Y. (2018). Depression in medical students: current insights. *Adv. Med. Educat. Pract.* 9:323. 10.2147/AMEP.S137384 29765261 PMC5944463

[B46] Constitution of the world health organization (1946). Constitution of the world health organization. *Am. J. Public Health Nations Health* 36 1315–1323. 10.2105/ajph.36.11.1315 18016450 PMC1625885

[B47] Norvilitis J. M. Mao Y. (2013). Attitudes towards credit and finances among college students in China and the United States. *Int. J. Psychol.* 48 389–398. 10.1080/00207594.2011.645486 22397472

[B48] Oxford R. L. (1993). Gender differences in styles and strategies for language learning: what do they mean? Should we pay attention. *Strategic Interact. Lang. Acquisit.* 1993 541–557.

[B49] Pavot W. Diener E. Suh E. (1998). The temporal satisfaction with life scale. *J. Personal. Assessm.* 70 340–354. 10.1207/s15327752jpa7002_11

[B50] Polit D. F. Beck C. T. (2006). The content validity index: are you sure you know what’s being reported? Critique and recommendations. *Res. Nurs. Health* 29 489–497. 10.1002/nur.20147 16977646

[B51] Powell-Young Y. M. (2012). Household income and spiritual well-being but not body mass index as determinants of poor self-rated health among African American adolescents. *Res. Nurs. Health* 35 219–230. 10.1002/nur.21473 22456912 PMC3348349

[B52] Puthran R. Zhang M. W. Tam W. W. Ho R. C. (2016). Prevalence of depression amongst medical students: a meta-analysis. *Med. Educ.* 50 456–468. 10.1111/medu.12962 26995484

[B53] Rath T. Harter J. K. Harter J. (2010). *Wellbeing: The Five Essential Elements.* New York, NY: Simon and Schuster.

[B54] Renshaw T. L. (2018). Psychometrics of the revised college student subjective wellbeing questionnaire. *Can. J. School Psychol.* 33 136–149. 10.1186/s12913-016-1423-5 27409075 PMC4943498

[B55] Ryan R. M. Huta V. Deci E. L. (2008). Living well: a self-determination theory perspective on eudaimonia. *J. Happ. Stud.* 9 139–170. 10.1007/s10902-006-9023-4

[B56] Ryff C. D. Singer B. (1998). The contours of positive human health. *Psychol. Inquiry* 9 1–28. 10.1207/s15327965pli0901_1

[B57] Sautman B. (2010). Scaling back minority rights: the debate about China’s ethnic policies. *Stan. J. Int.* 46:51.

[B58] Self A. Statistics O. (2014). *Measuring National Wellbeing: Life in the UK 2012.* Newport: ONS.

[B59] Shim S. Barber B. L. Card N. A. Xiao J. J. Serido J. (2010). Financial socialization of first-year college students: the roles of parents, work, and education. *J. Youth Adolesc.* 39 1457–1470. 10.1007/s10964-009-9432-x 20938727

[B60] Shimazu A. Schaufeli W. B. (2009). Is workaholism good or bad for employee well-being? The distinctiveness of workaholism and work engagement among Japanese employees. *Ind. Health* 47 495–502. 10.2486/indhealth.47.495 19834258

[B61] Shimazu A. Schaufeli W. B. Kubota K. Kawakami N. (2012). Do workaholism and work engagement predict employee well-being and performance in opposite directions? *Ind. Health* 50 316–321. 10.2486/indhealth.ms1355 22673364

[B62] Smale B. Hilbrecht M. (2014). *Canadian Index of Well-Being.* Berlin: Springer Netherlands, 10.1007/978-94-007-0753-5_253

[B63] Stewart S. M. Lam T. Betson C. Wong C. Wong A. (1999). A prospective analysis of stress and academic performance in the first two years of medical school. *Med. Educat.* 33 243–250. 10.1046/j.1365-2923.1999.00294.x 10336754

[B64] Taylor A. W. Kelly G. Dal Grande E. Kelly D. Marin T. Hey N. (2017). Population levels of wellbeing and the association with social capital. *BMC Psychol.* 5:23. 10.1186/s40359-017-0193-0 28673334 PMC5496434

[B65] Taylor J. M. (2021). Coefficient Omega. *J. Nurs. Educ.* 60 429–430. 10.3928/01484834-20210722-02 34346817

[B66] Tennant R. Hiller L. Fishwick R. Platt S. Joseph S. Weich S. (2007). The Warwick-Edinburgh Mental Well-being Scale (WEMWBS): development and UK validation. *Health Qual. Life Outcomes* 5:63. 10.1186/1477-7525-5-63 18042300 PMC2222612

[B67] UNESCO (2019). *Education.* Available online at: https://www.unesco.org/en

[B68] Upadyaya K. Salmela-Aro K. (2013). Development of school engagement in association with academic success and well-being in varying social contexts. *Eur. Psychol.* 18 136–147. 10.3310/hsdr04330 27905805

[B69] Veenhoven R. (2001). *Quality-of-Life and Happiness: Not Quite the Same.* Rotterdam: Erasmus University Rotterdam.

[B70] Walsemann K. M. Gee G. C. Gentile D. (2015). Sick of our loans: student borrowing and the mental health of young adults in the United States. *Soc. Sci. Med.* 124 85–93. 10.1016/j.socscimed.2014.11.027 25461865

[B71] Wang P. Chu P. Wang J. Pan R. Sun Y. Yan M. (2020). Association between job stress and organizational commitment in three types of Chinese university teachers: mediating effects of job burnout and job satisfaction. *Front. Psychol.* 11:576768. 10.3389/fpsyg.2020.576768 33132985 PMC7578428

[B72] Weyns T. Colpin H. Verschueren K. (2021). The role of school-based relationships for school well-being: how different are high- and average-ability students? *Br. J. Educ. Psychol.* 91 1127–1145. 10.1111/bjep.12409 33476050

[B73] Wheaton B. Muthen B. Alwin D. F. Summers G. F. (1977). Assessing reliability and stability in panel models. *Sociol. Methodol.* 8 84–136. 10.2307/270754

[B74] WHO (2016). *Burning Opportunity: Clean Household Energy for Health, Sustainable Development, and Wellbeing of Women and Children.* Geneva: WHO.

[B75] Wilson W. R. (1967). Correlates of avowed happiness. *Psychol. Bull.* 67:294. 10.1037/h0024431 6042458

[B76] Zeng W. Chen R. Wang X. Zhang Q. Deng W. (2019). Prevalence of mental health problems among medical students in China: a meta-analysis. *Medicine* 98:e15337. 10.1097/md.0000000000015337 31045774 PMC6504335

[B77] Zhang Y. Carciofo R. (2021). Assessing the wellbeing of Chinese university students: validation of a Chinese version of the college student subjective wellbeing questionnaire. *BMC Psychol.* 9:69. 10.1186/s40359-021-00569-8 33933167 PMC8088553

[B78] Zhou L. Parmanto B. (2020). Development and validation of a comprehensive well-being scale for people in the university environment (Pitt Wellness Scale) using a crowdsourcing approach: cross-sectional study. *J. Med. Internet Res.* 22:e15075. 10.2196/15075 32347801 PMC7221649

[B79] Zhou S. (2021). The Chinese path of integration and development among all ethnic groups from a comparative perspective between China and the west. *Int. J. Anthropol. Ethnol.* 5 1–29.

