# Supplementary material for: Psychometric Properties of the Chinese Revision of the Pitt Wellness Scale for People in the University Environment
Source: Front Psychol. 2022 May 12;13:899880. doi: 10.3389/fpsyg.2022.899880 (PMC9134205; doi:10.3389/fpsyg.2022.899880)
Supplement: Supplementary file 1 [file Data_Sheet_1.docx]

**SUPPLEMENTARY INFORMATION**

**Appendix 1**

Rotated factor loadings of the exploratory factor analysis with 34 items from 958 study participants.

| Items | Factor1 | Factor2 | Factor3 | Factor4 | Factor5 |
| --- | --- | --- | --- | --- | --- |
| 1 |  |  |  | 0.661 |  |
| 2 |  |  |  | 0.574 |  |
| 3 |  |  |  | 0.682 |  |
| 4 |  |  |  | 0.795 |  |
| 6 |  |  |  | 0.545 |  |
| 7 |  | 0.634 |  |  |  |
| 8 |  | 0.489 |  |  |  |
| 9 |  | 0.652 |  |  |  |
| 10 |  | 0.700 |  |  |  |
| 11 |  | 0.616 |  |  |  |
| 12 |  | 0.663 |  |  |  |
| 13 |  |  | 0.610 |  |  |
| 14 |  |  | 0.688 |  |  |
| 15 |  |  | 0.606 |  |  |
| **16** | **0.439** |  | **0.513** |  |  |
| 17 |  |  | 0.697 |  |  |
| 18 |  |  |  |  | 0.610 |
| 19 |  |  |  |  | 0.741 |
| 21 |  |  |  |  | 0.718 |
| 22 |  | 0.682 |  |  |  |
| 23 |  | 0.681 |  |  |  |
| **24** | **0.405** | **0.693** |  |  |  |
| **25** | **0.409** | **0.659** |  |  |  |
| 26 |  | 0.446 |  |  |  |
| **27** | **0.660** | **0.420** |  |  |  |
| 28 | 0.652 |  |  |  |  |
| 29 | 0.628 |  |  |  |  |
| 30 | 0.604 |  |  |  |  |
| 31 | 0.755 |  |  |  |  |
| 32 | 0.696 |  |  |  |  |
| 33 | 0.800 |  |  |  |  |
| 34 | 0.776 |  |  |  |  |
| 35 | 0.774 |  |  |  |  |
| 36 | 0. 754 |  |  |  |  |

Bold are deleted items

**Appendix 2**

Results of the mean values and difference tests of different socio-demographic variables in various domains of well-being.

**Physical domain**

| **Characteristic** | **Mean (SD)** | **P-value Pairwise differences^a^** |
| --- | --- | --- |
| **ROLE** |  | **<0.001** |
| Student | 17.03(6.23) |  |
| Faculty and Staff | 12.73(5.72) |  |
| **Gender** |  | **<0.001** |
| Male | 14.69(6.13) |  |
| Female | 17.39(6.19) |  |
| **Only Children** |  | 0.114 |
| Yes | 17.12(6.46) |  |
| No | 16.66(6.13) |  |
| **Ethnic groups** |  | 0.343 |
| Han nationality | 16.78(6.25) |  |
| Ethnic minority | 17.13(6.37) |  |
| **Education background** |  | **<0.001** |
| Junior college or below (1) | 17.51(6.28) | **(1)＞(2),(3),(4)** |
| Bachelor’s degree (2) | 15.85(6.05) | **(2)＞(4)** |
| Master’s degree (3) | 13.26(5.90) |  |
| Doctoral degree or above (4) | 13.82(3.22) |  |
| **Subject** |  | **0.009** |
| Medicine | 17.16(6.10) |  |
| Non-medical | 16.13(6.75) |  |
| **Household income** |  | **0.041** |
| ≤CNY5000 (1) | 17.11(6.18) |  |
| CNY5001—10000 (2) | 16.80(6.18) | **(4)＜(1),(2)** |
| CNY10001—20000 (3) | 16.16(6.93) |  |
| ＞CNY20000 (4) | 15.12(6.67) |  |
| **Monthly living expenses**  **(students)** |  | 0.402 |
| ≤CNY1000 (1) | 16.80(6.32) |  |
| CNY1001—1500 (2) | 17.27(5.98) |  |
| CNY1501—2000 (3) | 16.83(6.35) |  |
| ＞CNY2000 (4) | 16.49(7.67) |  |
| **Grade(students)** |  | **<0.001** |
| Freshman | 17.01(6.05) |  |
| Sophomore | 18.15(6.51) | **(1)＞(3),(5)** |
| Junior | 13.16(6.75) | **(2)＞(1),(3),(5)** |
| Senior | 14.56(5.00) |  |
| First-year postgraduate | 13.71(5.53) |  |

Bold values correspond to statistically significant correlations (p <0.05)

^a^Pairwise differences were p<0.05 (Bonferroni corrected)

**Psychological domain**

| **Characteristic** | **Mean (SD)** | **P-value Pairwise differences^a^** |
| --- | --- | --- |
| **ROLE** |  | 0.092 |
| Student | 23.32(9.26) |  |
| Faculty and Staff | 21.53(c8.99) |  |
| **Gender** |  | 0.057 |
| Male | 22.44(10.04) |  |
| Female | 23.45(9.04) |  |
| **Only Children** |  | 0.074 |
| Yes | 23.29(9.68) |  |
| No | 23.22(8.95) |  |
| **Ethnic groups** |  | **0.017** |
| Han nationality | 23.49(9.26) |  |
| Ethnic minority | 22.19(9.15) |  |
| **Education background** |  | 0.197 |
| Junior college or below (1) | 23.50(9.35) |  |
| Bachelor’s degree (2) | 22.94(9.00) |  |
| Master’s degree (3) | 21.26(9.74) |  |
| Doctoral degree or above (4) | 22.45(6.62) |  |
| **Subject** |  | 0.330 |
| Medicine | 23.41(8.99) |  |
| Non-medical | 22.84(10.13) |  |
| **Household income** |  | **0.001** |
| ≤CNY5000 (1)  CNY5001—10000 (2)  CNY10001—20000 (3)  ＞CNY20000 (4) | 23.90(9.14)  23.04(9.14)  21.44(9.60)  20.38(10.37) |  |
|  |  | **(1)＞(3),(4)**  **(2)＞(3),(4)** |
|  |  |  |
| **Monthly living expenses**  **(students)** |  | **0.014** |
| ≤CNY1000 (1) | 23.29(9.26) |  |
| CNY1001—1500 (2) | 23.85(9.06) | **(2)＞(3)** |
| CNY1501—2000 (3) | 22.11(9.36) |  |
| ＞CNY2000 (4) | 22.16(10.51) |  |
| **Grade(students)** |  | 0.230 |
| Freshman | 23.16(9.03) |  |
| Sophomore | 24.19(9.67) |  |
| Junior | 21.84(10.97) |  |
| Senior | 25.44(12,52) |  |
| First-year postgraduate | 23.26(9.26) |  |

Bold values correspond to statistically significant correlations (p <0.05)

^a^Pairwise differences were p<0.05 (Bonferroni corrected)

**Social domain**

| **Characteristic** | **Mean (SD)** | **P-value Pairwise differences^a^** |
| --- | --- | --- |
| **ROLE** |  | 0.465 |
| Student | 8.52(3.80) |  |
| Faculty and Staff | 8.20(3.79) |  |
| **Gender** |  | 0.898 |
| Male | 8.53(4.03) |  |
| Female | 8.50(3.75) |  |
| **Only Children** |  | 0.432 |
| Yes | 8.33(3.48) |  |
| No | 8.57(3.75) |  |
| **Ethnic groups** |  | 0.216 |
| Han nationality | 8.56(3.80) |  |
| Ethnic minority | 8.28(3.80) |  |
| **Education background** |  | 0.778 |
| Junior college or below (1) | 8.49(3.80) |  |
| Bachelor’s degree (2) | 8.59(3.81) |  |
| Master’s degree (3) | 8.11(3.81) |  |
| Doctoral degree or above (4) | 8.82(3.79) |  |
| **Subject** |  | 0.207 |
| Medicine | 8.42(3.58) |  |
| Non-medical | 8.75(4.47) |  |
| **Household income** |  | **0.013** |
| ≤CNY5000 (1) | 8.77(3.85) |  |
| CNY5001—10000 (2) | 8.35(3.68) | **(1)＞(2),(3),(4)** |
| CNY10001—20000 (3) | 8.01(3.97) |  |
| ＞CNY20000 (4) | 7.73(3.81) |  |
| **Monthly living expenses**  **(students)** |  | 0.080 |
| ≤CNY1000 (1) | 8.60(3.87) |  |
| CNY1001—150 (2) | 8.68(3.74) |  |
| CNY1501—2000 (3) | 8.12(3.68) |  |
| ＞CNY2000 (4) | 8.07(4.45) |  |
| **grade(students)** |  | 0.195 |
| Freshman | 8.46(3.72) |  |
| Sophomore | 8.57(3.82) |  |
| Junior | 9.10(5.03) |  |
| Senior | 11.11(4.76) |  |
| First-year postgraduate | 8.40(3.81) |  |

Bold values correspond to statistically significant correlations (p <0.05)

^a^Pairwise differences were p<0.05 (Bonferroni corrected)

**Financial domain**

| **Characteristic** |  | **Mean (SD)** | **P-value Pairwise differences^a^** |
| --- | --- | --- | --- |
| **ROLE** |  |  | **0.004** |
| Student |  | 9.59(3.92) |  |
| Faculty and Staff |  | 8.28(3.65) |  |
| **Gender** |  |  | **<0.001** |
| Male |  | 8.85(3.92) |  |
| Female |  | 9.71(3.90) |  |
| **Only Children** |  |  | **0.007** |
| Yes |  | 9.24(4.03) |  |
| No |  | 9.73(3.82) |  |
| **Ethnic groups** |  |  | 0.999 |
| Han nationality |  | 9.53(3.81) |  |
| Ethnic minority |  | 9.53(4.34) |  |
| **Education background** |  |  | **<0.001** |
| Junior college or below (1) |  | 9.88(3.98) |  |
| Bachelor’s degree (2) |  | 8.96(3.70) | **(1)＞(2),(3)** |
| Master’s degree (3) |  | 7.92(3.71) | **(2)＞ (3)** |
| Doctoral degree or above (4) |  | 8.82(3.40) |  |
| **Subject** |  |  | **<0.001** |
| Medicine |  | 9.75(3.85) |  |
| Non-medical |  | 8.93(4.04) |  |
| **Household income** |  |  | **<0.001** |
| ≤CNY5000 (1) |  | 10.37(3.96) | **(1)＞(2),(3),(4)** |
| CNY5001—10000 (2) |  | 9.01(3.62) | **(2)＞(3),(4)** |
| CNY10001—20000 (3) |  | 7.87(3.87) |  |
| ＞CNY20000 (4) |  | 7.17(3.27) |  |
| **Monthly living expenses**  **(students)** |  |  | **<0.001** |
| ≤CNY1000 (1) |  | 10.41(4.12) | **(1)＞(2),(3),(4)** |
| CNY1001—1500 (2) |  | 9.64(3.71) | **(2)＞(3),(4)** |
| CNY1501—2000 (3) |  | 8.68(3.73) |  |
| ＞CNY2000 (4) |  | 8.34(4.64) |  |
| **grade(students)** |  |  | 0.053 |
| Freshman |  | 9.63(3.87) |  |
| Sophomore |  | 9.71(4.03) |  |
| Junior |  | 8.35(4.49) |  |
| Senior |  | 9.78(3.35) |  |
| First-year postgraduate |  | 8.54(3.18) |  |

Italics values correspond to statistically significant correlations (p <0.05)

^a^Pairwise differences were p<0.05 (Bonferroni corrected)

**Competent domain**

| **Characteristic** | **Mean (SD)** | **P-value Pairwise differences^a^** |
| --- | --- | --- |
| **ROLE** |  | **0.044** |
| Student | 25.21(9.83) |  |
| Faculty and Staff | 23.01(9.81) |  |
| **Gender** |  | 0.066 |
| Male | 24.28(10.11) |  |
| Female | 25.33(9.76) |  |
| **Only Children** |  | 0.245 |
| Yes | 24.80(10.03) |  |
| No | 25.34(9.70) |  |
| **Ethnic groups** |  | **0.004** |
| Han nationality | 25.43(9.75) |  |
| Ethnic minority | 23.77(10.10) |  |
| **Education background** |  | **<0.001** |
| Junior college or below (1) | 24.83(9.88) |  |
| Bachelor’s degree (2) | 25.91(9.68) | **(1)＞(2),(3),(4)** |
| Master’s degree (3) | 24.36(10.51) | **(2)＞(3)** |
| Doctoral degree or above (4) | 22.64(7.28) |  |
| **Subject** |  | **0.004** |
| Medicine | 25.53(9.34) |  |
| Non-medical | 23.66(11.28) |  |
| **Household income** |  | **<0.001** |
| ≤CNY5000 (1)  CNY5001—10000 (2)  CNY10001—20000 (3)  ＞CNY20000 (4) | 25.87(9.59)  24.94(9.84)  23.23(10.52)  20.58(10.02) |  |
|  |  | **(1)＞(2),(3),(4) (2)＞(3),(4)** |
|  |  |  |
| **Monthly living expenses**  **(students)** |  | **<0.001** |
| ≤CNY1000 (1) | 25.26(9.92) | **(1)＞(2),(3),(4)** |
| CNY1001—1500 (2) | 25.70(9.45) | **(2)＞(3),(4)** |
| CNY1501—2000 (3) | 24.46(10.25) |  |
| ＞CNY2000 (4) | 22.72(11.15) |  |
| **grade(students)** |  | 0.053 |
| Freshman | 25.23(9.78) |  |
| Sophomore | 25.35(9.62) |  |
| Junior | 23.03(12.34) |  |
| Senior | 26.78(9.03) |  |
| First-year postgraduate | 26.00(9.73) |  |

Bold values correspond to statistically significant correlations (p <0.05)

^a^Pairwise differences were p<0.05 (Bonferroni corrected)

**Appendix 3**

Chinese Revision of the Pitt Wellness Scale—Chinese

身体领域

1、早晨醒来时，我感觉得到了休息。

2、每周，我都要适度锻炼至少30分钟（比如：快步走、用每小时慢于10英里的速度骑自行车、打网球、跳交谊舞）。

3、鉴于我的健康状况，我尽可能多地进行锻炼。

4、通常，我有足够的精力做日常活动。

5、最近，我胃口很好。

心理领域

6、我对自己的生活质量大体上是满意的。

7、基本上，我是自我认可的。

8、我对未来充满希望。

9、我觉得可以控制自己的情绪。

10、我相信生活是由自己创造的。

11、如果我的第一个计划失败，我愿意接受新的机会。

12、我觉得我的人生是有意义的。

13、在困难时期，我感受到了内在和/或精神的力量。

14、我的个人信仰（宗教与否）帮助我应对生活中的困难。

社交领域

15、我生活在一个安全的社区。

16、我遇到好事时，会和我的家人和/或朋友分享这种经历。

17、我能满足依赖我的人的需求，我对自己的这种能力感到满意。

18、生活中有关心我的人。

财务领域

19、如果意外发生了超出平均水平的支出，我的财务状况仍然稳定。

20、如果需要的话，会有人帮我处理财务问题。

21、我的收入/生活费足以满足我目前的需求。

能力领域

22、我对完成工作/未来工作职责所需的时间感到满意。

23、我的雇主，领导，学校，老师给我提供很多职业发展机会。

24、和同事/未来同事一起工作我感到很舒服。

25、我能很好平衡我的工作/未来的工作和生活。

26、我的工作/未来从事的职业稳定性很高。

27、我对自己的工作/学习质量很满意。

28、我很清楚自己的知识优势。

29、我可以依靠自己的天赋和技能处理突发情况。

30、我对自己的决策能力很满意。

**Appendix 4**

Chinese Revision of the Pitt Wellness Scale

**Physical domain**

1. I feel rested when I wake up in the morning.

2. Each week, I exercise moderately for at least 30 minutes (for instance, walking briskly, bicycling slower than 10 miles per hour, playing tennis, and ballroom dancing).

3. Because of my health status, I am physically able to exercise as much as I would like to.

4. I usually have enough energy for everyday activities.

5. My appetite has been good recently.

**Pshchological domain**

6. I am generally satisfied with my quality of life.

7. I am generally self-accepting.

8. I feel hopeful about the future.

9. I feel that I have control over my emotions.

10. I believe that life is what you make it.

11. I am open to new opportunities if my first plan does not work out.

12. I feel that my life is meaningful.

13. I feel inner and/or spiritual strength in difficult times.

14. My personal beliefs (religious or not) help me to cope with difficulties in life.

**Social domain**

15. I am living in a safe community.

16. When something good happens to me, I share the experience with my family and/or friends.

17. I am satisfied with my ability to meet the needs of people who depend on me.

18. I have people in my life who care about me.

**Financial domain**

19. If I incur an unexpected above average expense, I would still be stable financially.

20. I have someone to help with my financial affairs, if needed.

21. My income/ living expenses is adequate for my current needs.

**competent domain**

22. I am/ will be satisfied with the amount of time required by my job / future job duties.

23. My employers, leaders, teachers, or universities provide me many career development opportunities.

24. I feel/ will feel comfortable working with my colleagues / future colleagues.

25. My work / future [occupation](javascript:;) and life are/ will be well-balanced.

26. I think my job / future [occupation](javascript:;) security is high.

27. I am satisfied with the quality of my work/study.

28. I am aware of my intellectual strengths.

29. I can rely upon my talents and skills to handle unexpected situations.

30. I am satisfied with my ability to make decisions.
